# New Publications

**Published:** 1876-06

**Authors:** 


McComber's Tracks: An Appeal to Fathers and Mothers to save their Children's feet from Deformity. By Joel McComber, Inventor and Patentee of the McComber Last; also of the McComber Patent Glove-Fitting Boots and Shoes. New York, 1876.

This little work is written in the usual earnest vein of the enthusiastic inventor of the reformed system of constructing boots and shoes with strict reference to the anatomy of the foot. It is, as its title would indicate, less a discussion of the methods employed by him than an earnest recital of the miseries wrought upon mankind by the distorting influences of the clothing heretofore supplied for the tender feet of children. This little book admirably supplements the paper read by Mr. McComber before the polytechnic branch of the American Institute at Cooper Union last year. In that address the theory of the improvements of the author were set forth and made clear by several diagrams. The present work explains the necessity of beginning the work of reform in early life, when most of the serious deformities are initiated. A paragraph will show the general tone of the work:

"It behooves parents to start right, as well in the physical as in the moral development of their little ones. If they would have their children walk uprightly as men and women, let them be as careful how they distort their feet as they are how they permit their morals to be distorted. The errors of youth may be repented of; the deformity remains. To begin right with the child is the duty of the parent, because the child is not able to exercise intelligent judgment. With a start in the right direction all things are possible; but with a deformity commenced almost at the cradle, absolute relief is difficult and uncertain. The Chinaman who compresses the feet of his infant daughter is scarcely more cruel, and is guilty of hardly greater barbarity, than the American father who suffers the tender feet of his children to be practically ruined for life by forcing them into the nameless abominations which are generally considered respectable foot coverings."

Mr. McComber is clearly accomplishing a work which has been wrought by no other maker of foot-gear in any land. He alone, as we believe, of all the hundreds of thousands of the followers of St. Crispin, has made the anatomy of the human foot a profound study. It is probably fair to say that no other bootmaker in America, if, indeed, on the face of earth, has gained the knowledge requisite to complete success in his art, by the study of practical anatomy under competent instructors. He has fitted himself for the task which he has set before himself by the study of anatomy, by the dissection of human feet, perfect and deformed, and has thus familiarized himself with the diseases and distortions to which the ill-clothed feet of humanity are subject. With the knowledge thus acquired, he sets out to apply the remedy? He finds this remedy in a method of clothing the feet peculiarly his own. He sees that most of the suffering and all of the dis tortions of our feet arise from the errors of those who clothe these deli cate organs. He discovers that a multitude of diseases not of the feet are induced by the same cause. Naturally, the remedy applied is a radical one. It is radical in results, although it is not altogether apparent at first sight. Heretofore, the so-called "anatomical bootmakers" have fairly outraged all taste by the shape given to their shoes. Mr. McComber offers a very graceful shoe, as well as a perfectly comfortable one, yet he does not compress the foot; he gives its delicate network of muscles and nerves free space and a symmetrical covering. "McComber's Tracks"—the tracks made by the thousands of his boots and shoes now worn—are seen on every side. The encomimus passed upon his goods are uttered by a vast multitude. His last appeal to parents will not pass unheeded, because of the fact that he stands absolutely at the head of his profession, and is clearly to-day the best authority on this continent upon the subject of which he writes.

